# Quaternion-Based Robust Attitude Estimation Using an Adaptive Unscented Kalman Filter

**DOI:** 10.3390/s19102372

**Published:** 2019-05-23

**Authors:** Antônio C. B. Chiella, Bruno O. S. Teixeira, Guilherme A. S. Pereira

**Affiliations:** 1 Graduate Program in Electrical Engineering, Universidade Federal de Minas Gerais, Belo Hotizonte 31270-901, Brazil; acbchiella@ufmg.br (A.C.B.C.); brunoot@ufmg.br (B.O.S.T.); 2 Department of Mechanical and Aerospace Engineering, West Virginia University, Morgantown, WV 26506-6070, USA

**Keywords:** unit quaternion, unscented Kalman filter, MARG sensor, adaptive filtering

## Abstract

This paper presents the Quaternion-based Robust Adaptive Unscented Kalman Filter (QRAUKF) for attitude estimation. The proposed methodology modifies and extends the standard UKF equations to consistently accommodate the non-Euclidean algebra of unit quaternions and to add robustness to fast and slow variations in the measurement uncertainty. To deal with slow time-varying perturbations in the sensors, an adaptive strategy based on covariance matching that tunes the measurement covariance matrix online is used. Additionally, an outlier detector algorithm is adopted to identify abrupt changes in the UKF innovation, thus rejecting fast perturbations. Adaptation and outlier detection make the proposed algorithm robust to fast and slow perturbations such as external magnetic field interference and linear accelerations. Comparative experimental results that use an industrial manipulator robot as ground truth suggest that our method overcomes a trusted commercial solution and other widely used open source algorithms found in the literature.

## 1. Introduction

With the rise of small flying vehicles, also known as drones, small and inexpensive attitude and heading reference systems (AHRS) have populated the market. For such systems, it is common to estimate the orientation by combining information from magnetic, rate and gravity (MARG) sensors, also known as inertial measurement unit (IMU), usually composed of micromechanical (MEMS) three-axis gyroscope and accelerometer and a three-axis magnetometer [1]. The standard approach for attitude estimation is to compute the three components of inertial orientation by integrating the gyroscope measurements, and use the gravity projection and heading angle estimated by the accelerometers and magnetometers to correct the angles computed with the gyro. Although theoretically simple, naive implementations of this approach may not be precise because magnetometer measurements are easily influenced by ferrous material in the vicinity, and accelerometers measure not only the gravitational direction but also its linear acceleration. In these cases, it is difficult to dissociate magnetic field perturbation and linear acceleration from both the magnetic field of the earth and gravity to compute the attitude accurately, which can lead to poor estimates [2,3]. Since accurate attitude estimation is a crucial task for a variety of applications, such as human motion tracking [4,5], augmented reality [6], satellite control [7] and navigation and control of aerial [8], and sub-aquatic vehicles [9], the main objective of this paper is to present a solution that deals with external in internal sensor disturbance and interference. This paper extends the results of our conference paper [10]. We provide a more detailed mathematical description of our method and provide a whole new set of experiments. We also added several new figures that illustrate the ability of our method to estimate attitude and also some characteristics of the sensors, such as bias.

It is usually very common and useful to represent attitude by a combination of three successive rotations, such as Euler angles. Although intuitive, Euler angles exhibit singularity in their kinematic description, a situation known as gimbal lock. Alternatively, unit quaternion and direct cosine representations avoid such singularities. Between the two, unit quaternion is preferred due to its minimal representation and computational efficiency [11]. However, the unit quaternion representation does not pertain to the Euclidean space, meaning that weighted sum and subtraction operations, common to most sensor fusion methodologies, may violate the unit norm constraint of the quaternion. This paper proposes a solution to this problem in situations where the uncertainty of the measurements may change over time.

There are many approaches to estimate attitude using the quaternion representation. Among the stochastic approaches, techniques based on the Kalman filter (KF) and its variations for non-linear systems are the most common [12,13]. To overcome the Euclidean systematization using the original KF, an indirect form of the KF, called multiplicative extended Kalman filter (MEKF) [14], was proposed. This approach is valid only for small estimation errors. For large errors, algorithms based on the unscented Kalman filter (UKF) as the unscented quaternion estimator (USQUE) [15] may yield better results. Since unit quaternions are constrained to the nonlinear Riemannian manifold, using its *logarithm* and *exponential* maps, as in Refs. [11,16], can better handle its properties. A more general formulation of UKF for unit quaternion can be found in Ref. [17]. However, these algorithms do not explore the time-varying measurement uncertainty. If, for example, external disturbance affects the measurement of the magnetic field, the filter estimate will be severely damaged.

The classical way of handling the temporal variation of uncertainty is through adaptive filters, in which the statistical parameters that characterize the uncertainty are jointly estimated with the system states. In this context, approaches based on the techniques referred to as covariance matching (CM) [18], interacting multiple models (IMM) [19] and covariance scaling (CS) [20], were investigated. Among these methods, the covariance matching approaches yield improved results in the estimation of the covariance matrix for Gaussian distribution, if compared to the CS approach. CM also presents a greater simplicity if compared to approaches based on multiple models. However, in the presence of outliers, its performance can be damaged. In this context, median-based approaches can be combined to mitigate the outlier influence [21,22]. It is important to point out that, like the KF, these adaptive approaches also belong to Euclidean systematization, thus requiring modifications to be used with unitary quaternions.

In Ref. [23], the authors present an adaptive UKF for attitude estimation. The adaptive part of the algorithm is based on a covariance scaling approach, which adapts the covariance matrix if a fault is detected by a chi-square test. The main shortcoming of this approach is that the chi-square test may fail for slow varying faults, keeping the covariance matrix unchanged. In addition, the attitude is parameterized by Euler angles, being vulnerable to gimbal-lock. Based on the algorithms shown in Refs. [15,23], an adaptive UKF for attitude represented in quaternions is presented in Ref. [24]. In Ref. [25], a inflated covariance method based on multiplicative EKF (MEKF) is proposed to compensate the undesired effects of magnetic distortion and body acceleration. In Ref. [26], the Hidden Markov Model (HMM) is combined with MEKF to estimate the observation covariance matrix, thus compensating the undesired effects of magnetic distortion and linear acceleration. In spite of demonstrated good performance with numerically simulated cases [25] and actual data [26], these algorithms suffer the same limitation of MEKF, which is restricted to small estimate errors.

In contrast to KF-based approaches, which adopt a probabilistic determination of the modeled state, complementary filters (CF) are based on frequency analysis, being simplistic and usually computationally more efficient. In Ref. [27], the authors proposed an explicit complementary filter (ECF) for orientation estimation. Such a filter uses a proportional-integral (PI) controller to estimate the bias of the gyro. In Ref. [28], the authors present a computationally efficient gradient descent algorithm given measurements from a MARG sensor. The proposed algorithm has low computational cost and is able to reduce the effect of the magnetic disturbance. The problem of this algorithm is that the orientation estimated using accelerometers suffers the influence of magnetic disturbances due to the coupling in the gradient descent algorithm used. In Ref. [29], quaternion measurement is computed as the composition of two algebraic quaternions, mitigating the influence of magnetic distortion. Adaptive gains are used to reduce the estimation error during high dynamic motion. Instead of using adaptive factors, in Ref. [30], an air data sensor (ADS) is used to compensate for the linear acceleration disturbance in the MARG sensor. Although interesting, this solution requires a minimum system velocity, making its application limited.

In this scenario, we propose an adaptive algorithm based on UKF for attitude estimation using quaternion representation. In contrast to EKF, UKF-based approaches can better handle the high order nonlinear terms that can appear when the attitude error is large. The unit norm constraint of the quaternion is ensured by the use of a rotation vector parameterization. Differently from quaternion error approximation used on USQUE, this parameterization better handles the unit quaternion properties, such as the Riemannian metric [31]. The main difference between our work and classical approaches, such as MEKF and USQUE, is that it handles online the time-varying measurement uncertainty. For this, the covariance matching approach is used to update the observation covariance matrix online. Although CM can usually estimate well the measurement covariance matrix, the presence of outliers can damage the estimation. To minimize the effect of measurement outliers, we propose to combine the adaptive filter with a Hampel identifier, which compares the median deviation and the median absolute deviation (MAD) to identify an outlier. Our approach only uses the MARG sensor as an information source, and does not need extra information, such as ADS. Thus, *the main contribution of this paper* is the Quaternion-based Robust Adaptive Unscented Kalman Filter (QRAUKF) algorithm for attitude estimation. This algorithm is robust to fast and slow perturbations on both accelerometers and magnetometers and, to the best of authors’ knowledge, is the first one with such characteristics that precisely and consistently represent the attitude using quaternions. The proposed algorithm is tested with real experimental data collected from a MARG sensor. The performance of the proposed algorithm is confronted against the non-adaptive UKF, the open source algorithm based on complementary filter proposed in Ref. [32] and the commercial algorithm embedded in the MARG device used in our experiments, which was executed using a manipulator robot for validation purposes. A Matlab implementation of the algorithm is freely provided by the authors.

The rest of this paper is organized as follows. A problem formulation is presented in the next section. The theoretical basis for our contribution is in Section 3 and Section 4. The proposed algorithm is presented in Section 5 and is experimentally evaluated in Section 6. Finally, conclusions are presented in Section 7.

## 2. Problem Statement

This paper addresses the problem of attitude estimation of a rigid body moving in a three dimensional space. This problem basically consists in determining the three degrees-of-freedom orientation information of the body, independently of its current velocity and/or acceleration. Among the common representations for attitude, we chose to use unit quaternions, since it is a free-of-singularities and compact representation. To estimate the attitude, we assume that rigid body is attached to inertial and magnetic sensors, namely 3-axis accelerometer measuring acceleration in the three main body axis, $a_{x,m}$, $a_{y,m}$, and $a_{z,m}$; 3-axis magnetometer measuring the magnetic fields $b_{x,m}$, $b_{y,m}$, and $b_{z,m}$; and 3-axis gyroscope measuring the angular velocities $\omega_{x,m}$, $\omega_{y,m}$, and $\omega_{z,m}$. The accelerometers measure the gravity acceleration, which is indeed the part used for attitude estimation, along with all other accelerations to which the body is subjected, which act as perturbations for the movement. On the other hand, magnetometers measure the magnetic field of the earth, useful in the estimation process, and other unknown fields around the body. Measured accelerations and magnetic fields are contaminated with zero mean random noise. Angular velocity information, besides random noise, is also subjected to a time varying bias. Thus, our problem is to estimate the unit quaternion $e=[e_{1},e_{2},e_{3},e_{4}]$, given the vector of measurements $\left[a_{x,m},a_{y,m},a_{z,m},b_{x,m},b_{y,m},b_{z,m},\omega_{x,m},\omega_{y,m},\omega_{z,m}\right]$, which is corrupted with noise and external disturbances, as mentioned before.

**Figure 1:**
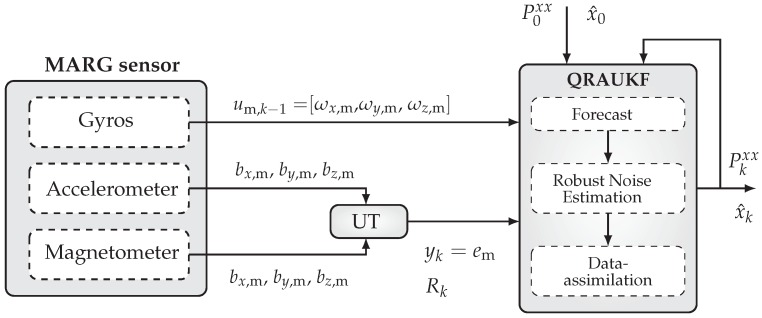
The architecture of the quaternion-based robust adaptive Kalman filter. In the left, the MARG sensor provides the measurement information. The filtering algorithm uses the gyros measurement $\omega_{m}\in R^{3}$ in the *forecast* step. The UT block is used to propagate the accelerometer $a_{m}\in R^{3}$ and magnetometer $b_{m}\in R^{3}$ measurements through a nonlinear function, computing a unit quaternion, used as a pseudo-measurement in the *robust noise estimation* and *data-assimilation* steps.

We solve the previous problem using a modified version of the Uncented Kalman Filter (UKF). A block diagram of our solution is shown in Figure 1. All blocks of this figure will be discussed in the following sections. We start by addressing attitude representation using unit quaternions and by presenting a new Unscented Transform (UT) for quaternions in the next section.

## 3. Unit Quaternion Operations

Unit quaternions form a four-dimensional algebra over the real numbers and can be used to parametrize the rotation group SO(3). The set of unit quaternions, denoted by $H_{1}$, form a group under multiplication operation but not under addition operation [17]. This group is topologically a 3-sphere, denoted by $S^{3}$, embedded in the $R^{4}$.

The unit quaternion can be represented as $e=\left(v,\;n\right)\in H_{1}$, in which ∥e∥=1. Here v∈R is the real part and $n\in R^{3}$ is the imaginary part. Given a rotation θ and the unit vector *w*, the corresponding unit quaternion is $e=\left(\cos\left(\frac{\theta }{2}\right),\;\sin\left(\frac{\theta }{2}\right)w\right)$. The inversion unit quaternion operation is equal to its conjugate, given by $e^{-1}=e^{*}=\left(v,\;-n\right)$. The product ⊗ between quaternions is defined as $e_{a}⊗e_{b}≜\left(v_{a}v_{b}-n_{a}^{T}n_{b},\;v_{a}n_{b}+v_{b}n_{a}+n_{a}\times n_{b}\right)$, in which × denotes the cross product [33]. A vector $v\in R^{3}$ can be rotated by a unit quaternion *e*, which is given by $\left(0,\;u\right)=e\left(0,\;v\right)e^{*}$, where $u\in R^{3}$ is the rotated vector [11].

### 3.1. Euclidean Tangent Space and Rotation Vector Parametrization

The group $S^{3}$ is a Riemannian manifold, whose elements can be represented in a three-dimensional Euclidean *tangent space* $T_{e}S^{3}$. Many operations are defined in the Euclidean tangent space, such as the empirical mean and covariance. Furthermore, there are direct and inverse mappings between the manifold and its *tangent space*, $S^{3}⟷T_{e}S^{3}$, with *exponential* and *logarithm* functions, respectively.

Let $e=\left(v,\;n\right)$ be a unit quaternion and r=θw be a rotation vector representing a rotation θ about the unit axis *w*. The unit quaternion to *rotation vector* mapping, called *logarithm* mapping, is given by [17]:

(1) $$\begin{array}{ccc} r & = & \left\{\begin{array}{c} 2\arccos\left(v\right)\frac{n}{∥n∥},\;\mathrm{if}\;∥n∥\neq 0\;\mathrm{and}\;v\geq 0\;,\\ -2\arccos\left(-v\right)\frac{n}{∥n∥},\;\mathrm{if}\;∥n∥\neq 0\;\mathrm{and}\;v<0\;,\\ {\left[0\right]}_{3\times 1},\;\mathrm{if}\;∥n∥=0\;, \end{array}\right. \end{array}$$

where the quaternion antipodal ambiguity e=−e is treated by checking the signal of *v*.

The inverse mapping, called *exponential* mapping, is [17]:

(2) $$\begin{array}{ccc} e & = & \left\{\begin{array}{c} \left(\cos\left(\frac{∥r∥}{2}\right),\;\sin\left(\frac{∥r∥}{2}\right)\frac{r}{∥r∥}\right)\;,\;\mathrm{if}\;∥r∥\neq 0\;,\\ (1,\;{\left[0\right]}_{3\times 1})\;,\;\mathrm{if}\;∥r∥=0\;. \end{array}\right. \end{array}$$

For brevity, *logarithm* (1) and *exponential* (2) mappings are written as e=R2Q(r) and r=Q2R(e), respectively.

### 3.2. Sum, Subtraction, and Weighted Mean Operations

We define now the operations of sum, subtraction, and weighted mean for unit quaternion states. The difference between $e_{a}$ and $e_{b}\in H_{1}$ is defined as

(3) $$\begin{array}{c} e_{a}⊖e_{b}≜Q2R\left(e_{a}⊗e_{b}^{*}\right)\;. \end{array}$$

Similarly, for the Euclidean vectors $\xi_{a}$ and $\xi_{b}\in R^{n}$, this operation is giving by $\xi_{a}-\xi_{b}$.

The sum of a unit quaternion $e_{a}\in H_{1}$ and a rotation vector $r\in R^{3}$, is defined as

(4) $$\begin{array}{c} e_{a}⊕r≜R2Q\left(r\right)⊗e_{a}\;. \end{array}$$

In Euclidean space, this operation is $\xi_{a}+\xi_{b}$.

Lastly, the weighted mean operation for a set of unit quaternions $E=\{e_{i}\}$, $i≜1\ldots n_{w}$, is defined as

(5) $$\begin{array}{ccc} \hat{e} & ≜ & \mathrm{WM}\left(E,W\right)\;, \end{array}$$

where $W=\{w_{i}\}$ is a set of corresponding weights. The quaternion mean $\hat{e}\in H_{1}$ can be computed in a closed form by [34]

(6) $$\begin{array}{ccc} M & ≜ & \sum_{i=1}^{n_{w}}w_{i}e_{i}e_{i}^{T}, \end{array}$$

where $M\in R^{4\times 4}$, and the quaternion mean is the eigenvector of *M* corresponding to the maximum eigenvalue. Iterative algorithms can also be used, as the gradient descent algorithm presented in Refs. [10,11].

### 3.3. Quaternion Unscented Transform

The *unscented* transform (UT) is the main core of UKF. The UT approximates the mean $\hat{y}\in R^{m}$ and its covariance $P^{yy}\in R^{m\times m}$ of a random variable *y* obtained from the nonlinear transformation $y=h(x_{1},x_{2},c)$, where $x_{1}\in R^{n_{1}}$ and $x_{2}\in R^{n_{2}}$ are random variables with mean ${\hat{x}}_{1}$ e ${\hat{x}}_{2}$ and covariance matrices $P^{x_{1}x_{1}}\in R^{\left(n_{1}-1\right)\times \left(n_{1}-1\right)}$ and $P^{x_{2}x_{2}}\in R^{n_{2}\times n_{2}}$, respectively, and *c* a known deterministic variable. In addition, the random variable $x_{1}$ is composed by a unit quaternion part $x_{1,H}$ and a unconstrained Euclidean part $x_{1,E}$; thus $x_{1}≜{\left[x_{1,H}^{T}\;x_{1,E}^{T}\right]}^{T}$.

Now, we define the augmented state vector $\overset{˘}{x}\in R^{\overset{˘}{n}}$ as

(7) $$\begin{array}{ccc} \overset{˘}{x} & ≜ & {\left[\begin{array}{cc} x_{1}^{T} & x_{2}^{T} \end{array}\right]}^{T}, \end{array}$$

where $\overset{˘}{n}=n_{1}+n_{2}$, as well as the augmented covariance matrix $P^{\overset{˘}{x}\overset{˘}{x}}\in R^{\left(\overset{˘}{n}-1\right)\times \left(\overset{˘}{n}-1\right)}$

(8) $$\begin{array}{ccc} P^{\overset{˘}{x}\overset{˘}{x}} & = & \left[\begin{array}{cc} P^{x_{1}x_{1}} & {\left[0\right]}_{\left(n_{1}-1\right)\times n_{2}}\\ {\left[0\right]}_{n_{2}\times \left(n_{1}-1\right)} & P^{x_{2}x_{2}} \end{array}\right]. \end{array}$$

The UT is based on a set of deterministically chosen samples known as sigma points (SP). The sigma points $X_{j}\in R^{\overset{˘}{n}-1}$ and the associated weights $w_{j}$, $j=1,\ldots ,2(\overset{˘}{n}-1)$ can be chosen asx

(9) $$\begin{array}{ccc} X & = & \hat{\overset{˘}{x}}{\left[1\right]}_{1\times 2(\overset{˘}{n}-1)}⊕\sqrt{\overset{˘}{n}-1}\left[{\left(P^{\overset{˘}{x}\overset{˘}{x}}\right)}^{\frac{1}{2}}\;-{\left(P^{\overset{˘}{x}\overset{˘}{x}}\right)}^{\frac{1}{2}}\right]\;,\; \end{array}$$

(10) $$\begin{array}{ccc} w_{j} & = & \frac{1}{2(\overset{˘}{n}-1)}, \end{array}$$

where $X_{j}$ is the *j*th column of matrix $X\in R^{\left(\overset{˘}{n}-1\right)\times 2\left(\overset{˘}{n}-1\right)}$, ${\left(\cdot \right)}^{\frac{1}{2}}$ is the Cholesky square root operation, and ${\left[1\right]}_{1\times 2(\overset{˘}{n}-1)}\in R^{1\times 2(\overset{˘}{n}-1)}$ is a row vector with elements equal to one. Notice that the columns of the covariance matrix $P^{\overset{˘}{x}\overset{˘}{x}}$ can be seen as a perturbation variable, where the unit quaternion part is parameterized as a *rotation vector*, which means that the covariance matrix is defined in the *tangent space*, hence the $\overset{˘}{n}-1$ dimension. The SP (9) can be partitioned as

(11) $$\begin{array}{ccc} \left[\begin{array}{c} X^{x_{1}}\\ X^{x_{2}} \end{array}\right] & ≜ & X, \end{array}$$

where $X^{x_{1}}\in R^{\left(n_{1}-1\right)\times 2\left(\overset{˘}{n}-1\right)}$ and $X^{x_{2}}\in R^{n_{2}\times 2\left(\overset{˘}{n}-1\right)}$.

Then each sigma point $X_{j}$ is propagated through *h*:

(12) $$\begin{array}{ccc} Y_{j} & = & h\left(X_{j}^{x_{1}},X_{j}^{x_{2}},c\right), \end{array}$$

where $Y_{j}={\left[Y_{j,H}^{T}\;Y_{j,E}^{T}\right]}^{T}\in R^{n_{y}}$ is the *j*th column of the matrix $Y\in R^{n_{y}\times 2\left(\overset{˘}{n}-1\right)}$.

From (12), we obtain $\hat{y}$, $P^{yy}$ and $P^{x_{1}y}$ as

(13) $$\begin{array}{ccc} \hat{y} & = & \mathrm{WM}\left(Y,w\right), \end{array}$$

(14) $$\begin{array}{ccc} P^{yy} & = & \sum_{j=1}^{2(\overset{˘}{n}-1)}w_{j}\left(Y_{j}⊖\hat{y}\right){\left(Y_{j}⊖\hat{y}\right)}^{T}, \end{array}$$

(15) $$\begin{array}{ccc} P^{x_{1}y} & = & \sum_{j=1}^{2(\overset{˘}{n}-1)}w_{j}\left(X_{j}^{x_{1}}⊖{\hat{x}}_{1}\right){\left(Y_{j}⊖\hat{y}\right)}^{T}. \end{array}$$

At this point it is important to mention that Equations (9) and (13)–(15) differ from the one in the standard UT transform because they consider the quaternion operations previously defined in this section.

From now on, for notation simplicity, we define the quaternion unscented transformation as the function UT(·) comprising the set of Equations (9)–(15) as:

(16) $$\begin{array}{ccc} \left\{\hat{y},P^{yy},P^{xy}\right\} & = & \mathrm{UT}\left(\hat{\overset{˘}{x}},P^{\overset{˘}{x}\overset{˘}{x}},c,h\right)\;. \end{array}$$

where $\hat{\overset{˘}{x}}$ and $P^{\overset{˘}{x}\overset{˘}{x}}$ are given by Equations (7) and (8), respectively.

## 4. Mathematic Modeling

This section describes the discrete time dynamic model used by the filtering algorithm presented in this paper.

### 4.1. Kinematic Model of Attitude

Assuming that angular rates $\omega_{k}\in R^{3}$, measured by a 3-axis gyros from the input vector $u_{k}$ of the dynamic system, the discrete-time attitude model is given by [15]

(17) $$\begin{array}{ccc} \mathrm{vec}\left(e_{k}\right) & = & A_{k-1}\mathrm{vec}\left(e_{k-1}\right), \end{array}$$

where $\mathrm{vec}\left(\cdot \right):H_{1}\to R^{4}$ is an operator that takes the four coefficients of the unit quaternion and stacks them in a 4-vector, *k* denotes the discrete time, and

$$\begin{array}{ccc} A_{k-1} & ≜ & c\left(\frac{T}{2}\left\|\omega \right\|\right)I_{4\times 4}+\frac{T}{2}s\left(\frac{T}{2}\left\|\omega \right\|\right)\Omega \left(\omega \right)\;,\\ \Omega (\omega ) & ≜ & \left[\begin{array}{cccc} 0 & -\omega_{x} & -\omega_{y} & -\omega_{z}\\ \omega_{x} & 0 & \omega_{z} & -\omega_{y}\\ \omega_{y} & -\omega_{z} & 0 & \omega_{x}\\ \omega_{z} & \omega_{y} & -\omega_{x} & 0 \end{array}\right]\;. \end{array}$$

We assume that $u_{k}=\omega_{k}\;\in \;R^{3}$ is corrupted with random noise and bias terms, modeled as $u_{m,k}=u_{k}+\beta_{k}+q_{u,k}$, in which “m” denotes a measured variable, $u_{m,k}=\left[\omega_{x_{m}}\right.$ $\omega_{y_{m}}$ ${\left.\omega_{z_{m}}\right]}^{T}\in R^{3}$ are angular rates measured by a 3-axis gyros, $\beta_{k}=\left[\beta_{\omega_{x}}\right.$ $\beta_{\omega_{y}}$ ${\left.\beta_{\omega_{z}}\right]}^{T}\in R^{3}$ are bias terms, and $q_{u,k}∼N\left({\left[0\right]}_{3\times 1},\;Q_{u}\right)\;\in \;R^{3}$ is the input random noise. To directly use the measured inputs in (17), bias terms and random noise are estimated and subtracted from the measurement. Then,

(18) $$\begin{array}{ccc} u_{k} & = & u_{m,k}-\beta_{k}-q_{u,k}\;. \end{array}$$

A more general model for gyrometers is $u_{m,k}=\left(I_{3\times 3}+S\right)u_{k}+\beta_{k}+q_{u,k}$, where $S\in R^{3\times 3}$ denotes the matrix of scale factors and misalignment. This model is more appropriate for in-lab calibration [2]. Thus, in this paper, we assume that scale factors and misalignment have already been determined and compensated, and only the biases are estimated [35].

Due to bias, which may vary in time, the attitude estimated based on gyros measurements may suffer a temporal drift. To minimize this problem, bias terms $\beta_{k}$ are modeled as a random walk process,

(19) $$\begin{array}{ccc} \beta_{k} & = & \beta_{k-1}+q_{\beta ,k-1}, \end{array}$$

where $q_{\beta }∼N\left({\left[0\right]}_{3\times 1},\;Q_{\beta }\right)\;\in \;R^{3}$ and are jointly estimated with the other system states, yielding a *joint state vector* $x_{k}≜{\left[\mathrm{vec}\left(e_{k}\right)\;\;\beta_{k}^{T}\right]}^{T}\;\in \;R^{7}$.

Equations (17) and (19) compose the *process model*, which can be compactly presented as

(20) $$\begin{array}{ccc} x_{k} & = & f\left(x_{k-1},\;q_{k-1},\;u_{k-1},\;k-1\right), \end{array}$$

where *f* denotes a nonlinear function of previous state $x_{k-1}$, with input $u_{k-1}$, and process noise $q_{k-1}≜{\left[q_{u,k-1}^{T}\;\;q_{\beta ,k-1}^{T}\right]}^{T}$.

### 4.2. Observation Model

The observation model relates the components of state vector $x_{k}$ with the output measurement vector $y_{k}\in H_{1}$, defined as $y_{k}≜e_{m}$. Measurements are corrupted by random errors and modeled as $e_{m}=e_{k}⊕r_{k}$, where $r_{k}∼N\left({\left[0\right]}_{3\times 1},R_{k}\right)\in R^{3}$ is the measurement noise parameterized as a *rotation vector*. Therefore, the *observation model* may be written as

(21) $$\begin{array}{ccc} y_{k} & = & h\left(x_{k},\;r_{k},\;k\right)\;. \end{array}$$

In this paper, the measured acceleration $a_{m,k}={\left[a_{x}\;a_{y}\;a_{z}\right]}^{T}\in R^{3}$ and magnetic field $b_{m,k}={\left[b_{x}\;b_{y}\;b_{z}\right]}^{T}\in R^{3}$ are used to compute the unit quaternion $e_{m}\in H_{1}$. Assuming normalized measurements such that $∥a_{m,k}∥=1$ and $∥b_{m,k}∥=1$, the unit quaternion representing the body attitude can be computed as [13,29]:

(22) $$\begin{array}{ccc} e_{m}^{*}\left(a_{m},b_{m}\right) & = & e_{\mathrm{acc}}⊗e_{\mathrm{mag}}\;, \end{array}$$

where $e_{m}^{*}\left(a_{m},b_{m}\right)$ is the conjugate of $e_{m}\left(a_{m},b_{m}\right)$ and

(23) $$\begin{array}{ccc} e_{\mathrm{acc}} & = & \left\{\begin{array}{cc} \left(\lambda_{1},{\left[-\frac{a_{y}}{2\lambda_{1}}\;\frac{a_{x}}{2\lambda_{1}}\;0\right]}^{T}\right), & a_{z}\geq 0\\ \left(-\frac{a_{y}}{2\lambda_{2}},{\left[\lambda_{2}\;0\;\frac{a_{x}}{2\lambda_{2}}\right]}^{T}\right), & a_{z}<0, \end{array}\right. \end{array}$$

(24) $$\begin{array}{ccc} e_{\mathrm{mag}} & = & \left\{\begin{array}{cc} \left(\frac{\lambda_{3}}{\sqrt{2\Gamma }},{\left[0\;0\;\frac{\lambda_{3}}{\sqrt{2}\Gamma }\right]}^{T}\right), & l_{x}\geq 0\\ \left(\frac{l_{y}}{\sqrt{2}\lambda_{4}},{\left[0\;0\;\frac{\lambda_{4}}{\sqrt{2\Gamma }}\right]}^{T}\right), & l_{x}<0, \end{array}\right. \end{array}$$

where $\lambda_{1}=\sqrt{\left(a_{z}+1\right)/2}$, $\lambda_{2}=\sqrt{\left(1-a_{z}\right)/2}$, $\Gamma =l_{x}^{2}+l_{y}^{2}$, $\lambda_{3}=\sqrt{\Gamma +l_{x}\sqrt{\Gamma }}$, $\lambda_{4}=\sqrt{\Gamma -l_{x}\sqrt{\Gamma }}$ and $l_{m,k}={\left[l_{x}\;l_{y}\;l_{z}\right]}^{T}$ such that $\left(0,l_{m,k}\right)=e_{acc}^{*}\left(0,b_{m,k}\right)e_{acc}$.

Because these equations are nonlinear, the unscented transform, given in Equation (16), is used to propagate the measured acceleration error $r_{a,k}∼N\left({\left[0\right]}_{3\times 1},R_{a}\right)\in R^{3}$ and magnetic field error $r_{b,k}∼N\left({\left[0\right]}_{3\times 1},R_{b}\right)\in R^{3}$ through Equations (22)–(24). Then,

(25) $$\begin{array}{ccc} \left(y_{k},R_{k},-\right) & = & \mathrm{UT}\left({\left[a_{m}^{T}\;b_{m}^{T}\right]}^{T},\mathrm{diag}\left(R_{a},R_{b}\right),0,h_{\mathrm{ab}}\right), \end{array}$$

where diag(A,B) forms a block diagonal matrix with matrices *A* and *B*, and $h_{ab}\left(a_{m},b_{m}\right)\in H_{1}$ is given by Equation (22).

## 5. State Estimators

We assume that our dynamic system is modeled by the nonlinear Equations (20) and (21) in which, ∀k≥1, the known data are the measured output $y_{k}$ and input $u_{k-1}$. It is also assumed that process noise $q_{k-1}\in R^{n_{q}}$ and measurement noise $r_{k}\in R^{n_{r}}$ are mutually independent with covariance matrices $Q_{k-1}\in R^{n_{q}\times n_{q}}$ and $R_{k}\in R^{n_{r}\times n_{r}}$, respectively. The state estimation problem aims at providing approximations for the mean ${\hat{x}}_{k}=E\left[x_{k}\right]$ and covariance $P_{k}^{xx}=E\left[\left(x_{k}⊖{\hat{x}}_{k}\right){\left(x_{k}⊖{\hat{x}}_{k}\right)}^{T}\right]$ that characterize the *a posteriori* probability density function (PDF) $\rho (x_{k}|y_{1:k})$.

Due to the nonlinear characteristics of the model, our proposition is to use as the basis to our approach the unscented Kalman filter (UKF) [36]. In the standard form of the UKF, two problems arise when it is used to estimate attitude: (i) the UKF pertains to Euclidean systematization, therefore containing sum and weighting operations, which are not defined for unit quaternions; (ii) the output measured noise $r_{k}$ can have time-varying statistical properties, which can, in the worst case, lead to diverging estimates. Regarding (i), most of the issues are solved if the modified unscented transform presented in Section 3.3 is applied in the place of the standard one, as shown in Section 5.1. The solution of (ii) is our core contribution. We consider two events that may change the statistical properties of measured noise: a dynamic event, such as linear accelerations that mask the gravity vector projection measured by accelerometers; and a external influence, such as a ferromagnetic element that disturbs the Earth’s magnetic field measured by the magnetometers. The rejection of these perturbations is addressed in Section 5.2 and Section 5.3.

### 5.1. Quaternion-Based UKF

The UKF algorithm presented in this section is based on the ones shown in Refs. [11,17], which are slightly modified to encompass direct unit quaternion measurements and multiplicative noise in the process. Henceforth, the notation ${\hat{x}}_{k|k-1}$ indicates an estimate of $x_{k}$ at time *k* based on information available up to and including time k−1. Likewise, ${\hat{x}}_{k}$ indicates an estimate of $x_{k}$ at time *k* based on information available up to and including time *k*. Let the process noise be partitioned as $q_{k-1}≜{\left[q_{1,k-1}^{T}\;\;q_{2,k-1}^{T}\right]}^{T}\;\in \;R^{n_{q}}$ with covariance matrix $Q_{k-1}≜\mathrm{diag}\left(Q_{1,k-1},\;Q_{2,k-1}\right)\;\in \;R^{n_{q}\times n_{q}}$, where $q_{1,k-1}\;\in \;R^{n_{q}-n_{x}+1}$ is the noise nonlinearly related to the state vector and $q_{2,k-1}\;\in \;R^{n_{x}-1}$ is the linear partition of noise. To improve the numerical stability of the filter, additive noise is considered for all states [37].

Given these definitions, the modified quaternion-based unscented Kalman filter (QUKF) *forecast* step is given by

(26) $$\begin{array}{ccc} \left({\hat{x}}_{k|k-1},{\tilde{P}}_{k|k-1}^{xx},\emptyset \right) & = & \mathrm{UT}\left({\hat{\overset{˘}{x}}}_{k-1},P_{k-1}^{\overset{˘}{x}\overset{˘}{x}},u_{k-1},f\right)\;,\; \end{array}$$

(27) $$\begin{array}{ccc} P_{k|k-1}^{xx} & = & {\tilde{P}}_{k|k-1}^{xx}+Q_{2,k-1}, \end{array}$$

(28) $$\begin{array}{ccc} \left({\hat{y}}_{k|k-1},{\tilde{P}}_{k|k-1}^{yy},P_{k|k-1}^{xy}\right) & = & \mathrm{UT}\left({\hat{x}}_{k|k-1},P_{k|k-1}^{xx},0,h\right)\;, \end{array}$$

(29) $$\begin{array}{ccc} P_{k|k-1}^{yy} & = & {\tilde{P}}_{k|k-1}^{yy}+R_{k}, \end{array}$$

(30) $$\begin{array}{ccc} \nu_{k} & = & y_{k}⊖{\hat{y}}_{k|k-1}, \end{array}$$

where $\nu_{k}$ is the innovation. The augmented state vector ${\overset{˘}{x}}_{k-1}\in R^{\overset{˘}{n}}$ and the corresponding covariance matrix $P_{k-1}^{\overset{˘}{x}\overset{˘}{x}}\in R^{\overset{˘}{n}\times \overset{˘}{n}}$ are respectively given by

$$\begin{array}{ccc} {\overset{˘}{x}}_{k-1} & ≜ & {\left[\begin{array}{cc} x_{k-1}^{T} & q_{1,k-1}^{T} \end{array}\right]}^{T}\;,\\ P_{k-1}^{\overset{˘}{x}\overset{˘}{x}} & ≜ & \left[\begin{array}{cc} P_{k-1}^{xx} & {\left[0\right]}_{\left(n_{x}-1\right)\times \left(n_{q}-n_{x}+1\right)}\\ {\left[0\right]}_{\left(n_{q}-n_{x}+1\right)\times \left(n_{x}-1\right)} & Q_{1,k-1} \end{array}\right]\;,\; \end{array}$$

with $\overset{˘}{n}=n_{q}+1$.

The state estimate and error covariance matrix are updated using information from $y_{k}$ in the *data-assimilation* step, given by:

(31) $$\begin{array}{ccc} K_{k} & = & P_{k|k-1}^{xy}{\left(P_{k|k-1}^{yy}\right)}^{-1}, \end{array}$$

(32) $$\begin{array}{ccc} {\hat{x}}_{k} & = & {\hat{x}}_{k|k-1}⊕K\nu_{k}, \end{array}$$

(33) $$\begin{array}{ccc} P_{k}^{xx} & = & P_{k|k-1}^{xx}-K_{k}P_{k|k-1}^{yy}K_{k}^{T}. \end{array}$$

### 5.2. Adaptive Covariance Matrix

The uncertainty of measurements in the UKF is represented by covariance matrix $R_{k}$, which is usually constant. However, the measurement uncertainties can be time-varying. We then propose the use of innovation $\nu_{k}$ to adjust the measurement covariance matrix online through the covariance matching (CM) approach [18].

Based on the assumption that the observation covariance matrix $R_{k}$ is constant during a sliding sampling window with finite length *N*, the basic idea of CM is to make the innovation $\nu_{k}$ consistent with its covariance $E\left[\nu_{k}\nu_{k}^{T}\right]≜P_{k|k-1}^{yy}$. Notice that the innovation pertains to the three-dimensional Euclidean *tangent space*. Thus, the covariance of $\nu_{k}$ is estimated as based on the last *N* innovation samples as

(34) $$\begin{array}{c} E\left[\nu_{k}\nu_{k}^{T}\right]\approx \frac{1}{N}\sum_{j=k-N+1}^{k}\nu_{j}\nu_{j}^{T}. \end{array}$$

Notice that, the UKF (see Equation (29)) approximates the covariance by $E\left[\nu_{k}\nu_{k}^{T}\right]≜{\tilde{P}}_{k|k-1}^{yy}+R_{k}$, where ${\tilde{P}}_{k|k-1}^{yy}≜\sum_{j=1}^{2(n-1)}w_{j}{\tilde{y}}_{j,k|k-1}{\tilde{y}}_{j,k|k-1}^{T}$. Then, $R_{k}$ can be estimated by

(35) $$\begin{array}{ccc} {\hat{R}}_{k} & = & \frac{1}{N}\sum_{j=k-N+1}^{k}\nu_{j}\nu_{j}^{T}-{\tilde{P}}_{k|k-1}^{yy}. \end{array}$$

To avoid negative values due the subtraction operation in Equation (35), negative values in ${\hat{R}}_{k}$ are replaced by their correspondent value in the nominal covariance matrix $R_{0}$.

### 5.3. Outlier Rejection

Outliers are spurious data that contaminate the statistical distribution. The contaminated measurements deviate significantly from the normal observations, which directly reflects in the innovation value $\nu_{k}$, and, consequently, in the covariance estimated by CM.

The Hampel identifier [38] is an outlier identification method that is reported as extremely effective in practice [39]. Based on this approach, our contribution is to compute a gain $\lambda \in R^{n_{r}\times n_{r}}$ to be used as a multiplier that reduces the outlier influence in the estimation of the covariance matrix and also on the Kalman gain. This gain is a diagonal matrix, wherein each the diagonal is defined as

(36) $$\begin{array}{ccc} \lambda_{j,ii} & ≜ & \min\left(1,\;\frac{n_{\sigma }s_{i}}{|\nu_{j,i}-\mathrm{med}\left\{\nu_{j,i}\right\}|}\right), \end{array}$$

where $s_{i}=1.4826\;\mathrm{med}\{|\nu_{j,i}-\mathrm{med}\left\{\nu_{j,i}\right\}\left|\right\}$ is the median absolute deviation (MAD), $n_{\sigma }$ is the number of standard deviations (confidence region) by which the innovation sample must differ from the local median, med is the median operator, {·} is a moving window with size *N*, j≜k−N+1…k is an index for each element of the moving window, and *i* is the index of each element of the innovation vector.

### 5.4. Quaternion-Based Robust Adaptive Unscented Kalman Filter

By combining Equations (35) and (36) with the QUKF Equations (26)–(33), we then obtain a three step algorithm that we call quaternion-based robust adaptive unscented Kalman filter (QRAUKF). The first step is the *forecast* step, which is given by Equations (26)–(28) and (30). The second step is the *robust noise estimation* given by Equation (36), the estimate covariance

(37) $$\begin{array}{ccc} {\hat{R}}_{k} & = & \max\left(\frac{1}{N}\sum_{j=k-N+1}^{k}\lambda_{j}\nu_{j}{\left(\lambda_{j}\nu_{j}\right)}^{T}-{\tilde{P}}_{k|k-1}^{yy},R_{0}\right)\; \end{array}$$

and (29). The third and last step is the *data-assimilation* step, which is given by Equations (31) and (33), and

(38) $$\begin{array}{ccc} {\hat{x}}_{k} & = & {\hat{x}}_{k|k-1}⊕K\lambda_{k}\nu_{k}\;. \end{array}$$

## 6. Experimental Results and Discussion

In this section we compare the performance of the proposed QRAUKF algorithm to the QUKF, the complementary filter (CF) proposed in Ref. [32], and the commercial algorithm embedded in the MicroStrain 3DM-GX1${}^{®}$ IMU. We implemented QRAUKF using Matlab (Our code is available at https://bitbucket.org/coroufmg/raukf_cm).

**Figure 2:**
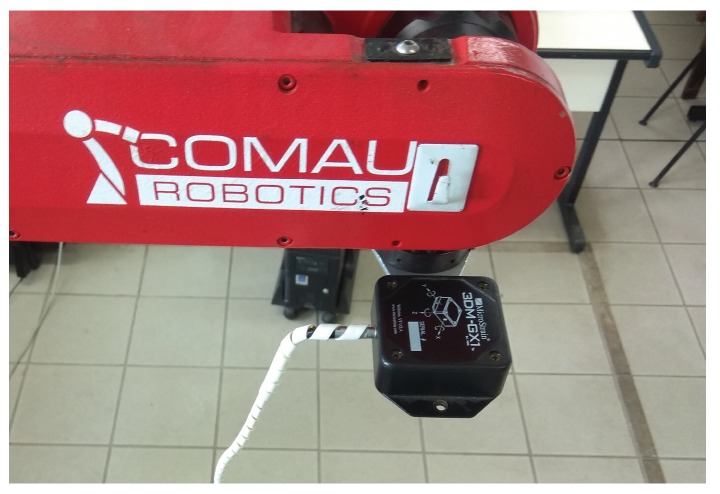
Experimental setup using the MicroStrain 3DM-GX1${}^{®}$ IMU and the Comau Smart Six${}^{®}$ robot.

Actual data was collected at 40 Hz from the IMU, which was mounted on the end effector of a Comau Smart Six${}^{®}$ manipulator, used to perform controlled movements and to provide accurate orientation information. Figure 2 illustrates our setup.

To set QUKF and QRAUKF, we have assumed that the covariance matrix $Q_{1,k-1}\in R^{3\times 3}$ is diagonal with elements related to the angular rates measured by the gyros. This matrix was estimated as $\sigma_{\omega }={\left[0.4584\;0.3724\;0.4927\right]}^{T}\;$deg/s. The additive noise of process was represented by the diagonal matrix $Q_{2,k-1}\in R^{6\times 6}$. This matrix is related to the attitude, parametrized as a rotation vector, and the bias terms of the gyros. The standard deviations were empirically set as $\sigma_{v}={\left[57.3\times 10^{-20}\right]}_{3\times 1}\;$deg and $\sigma_{\beta }={\left[57.3\times 10^{-9}\right]}_{3\times 1}\;$deg/s, for attitude and bias terms, respectively. The measured acceleration and magnetic field are propagated through the nonlinear function represented by Equations (22)–(24). Standard deviations of accelerometer and magnetometer are $\sigma_{a}={\left[0.0361\;0.0455\;0.0330\right]}^{T}$m/s${}^{2}$ and $\sigma_{m}={\left[0.0011\;0.00098\;0.00098\right]}^{T}$ Gauss [G], respectively. The nominal covariance matrix of measurements $R_{k}$ is computed by the UT. The sliding window size of RAUKF was empirically set to be N=20 samples, which represents a period of 0.5s during which the noise covariance is assumed to be constant, and the confidence region $n_{\sigma }=3$ standard deviations. CF has two parameters, the gain that quantifies the gyro measurement noise, set as β=0.007, and the gain that quantifies the bias terms, set as ζ=0.01. These values follows the authors recommendations [32]. The standard deviations $\sigma_{\omega }$, $\sigma_{a}$, and $\sigma_{m}$ were estimated from experimental data. For this, we have used a window of approximately 20s. During this calibration process, the MARG sensor was kept stationary (steady-state behavior). The tuning parameters were estimated before performing the main state estimation experiments, during which they remain unaltered. Observe that the measurement covariance matrix $R_{k}$ is updated online, in contrast with the process covariance matrices $Q_{1,k-1}$ and $Q_{2,k-1}$, that also remain unaltered. Although we used a simplistic parameter estimation approach, the parameterization seems to be appropriate, even for different experiments.

Five disturbance scenarios were evaluated: (i) abrupt and (ii) slow varying magnetic disturbances; (iii) linear accelerations; (iv) individual axis rotation about the origin; and (v) simultaneous axes rotations about the origin. The last two experiments, scenarios (iv)–(v), suffer linear accelerations due to the lever arm between the end effector of robot and IMU (Videos showing the experiments are found at: https://goo.gl/mtFSqG).

### 6.1. Magnetic Field Distortion

**Figure 3:**
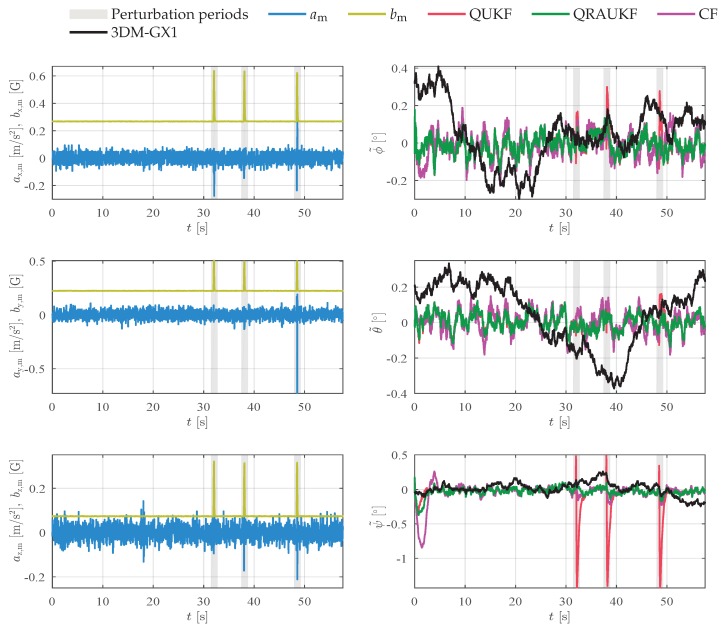
Results for abrupt magnetic disturbances experiment, scenario (i). In the left column, linear acceleration $a_{m}$ and magnetic field $b_{m}$ measurements, in the right column, the attitude error.

Heading estimation is performed by monitoring the Earth’s magnetic field with the magnetometer. Due to the proximity of ferrous or magnetic materials, the magnetic field can be locally distorted, which causes inaccurate estimates of heading angle. In our first experiment, the magnetic brakes of the robot manipulator are turned on and off a few times, thus causing an abrupt variation in the magnetic field that is perceived by the magnetometers. Due to the shaking caused by the release of the brakes, some spikes of acceleration also appear. Figure 3 shows the acceleration and magnetic field measurements, and the attitude estimation error for each axis. The disturbance periods are highlighted. Observe that the QUKF is more sensitive to outliers in the acceleration and magnetic field measurements, converging quickly to wrong estimates induced by inaccurate measurements. This behavior is expected, once QUKF does not have any treatment for abnormal measurements. In contrast, QRAUKF, CF, and 3DM-GX1 algorithms reject the outliers. Table 1 shows the Root Mean Square Error (RMSE) for this experiment in the column called “Abrupt magnetic”. Observe that QRAUKF performs better than other algorithms, with the smaller RMSE. In contrast, QUKF yields the largest RMSE indices.

**Figure 4:**
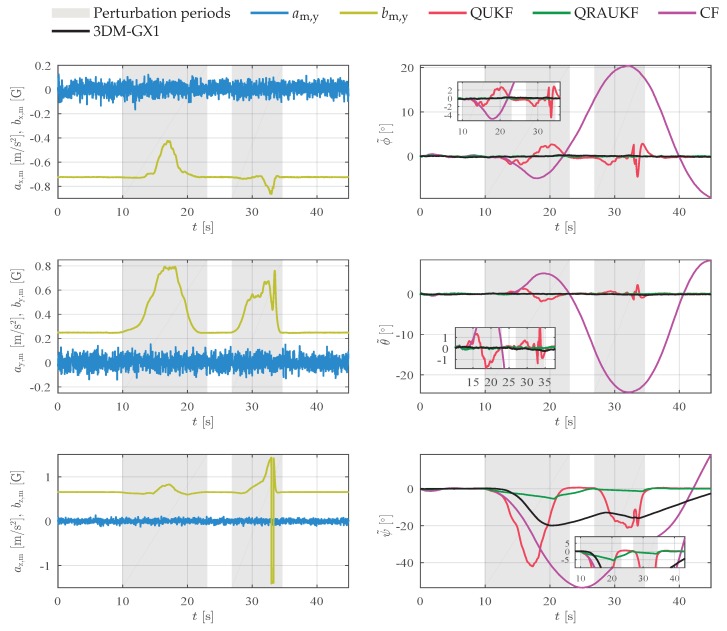
Results for slow magnetic disturbances experiment (second experiment), scenario (ii). In the left column, linear acceleration $a_{m}$ and magnetic field $b_{m}$ measurements, in the right column, the attitude error.

**Figure 5:**
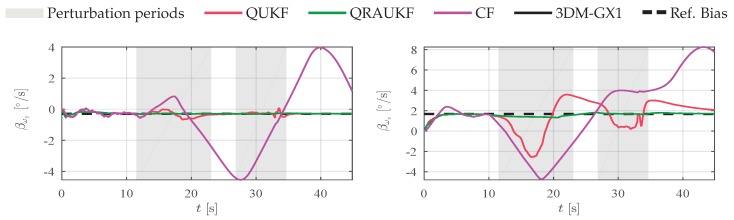
Bias estimate of angular rate $\omega_{y}$ and $\omega_{z}$, respectively, measured by the gyros for second experiment, scenario (ii).

In a second experiment, the magnetic field was artificially and slowly disturbed with a magnetic material. Figure 4 shows the acceleration and magnetic field measurements, and the attitude error for each axis. This kind of perturbation is usually difficult to detect and can damage the estimation. Notice that QRAUKF is less sensitive to the slow varying abnormal measurement. CF yields the worst results as shown by RMSE in Table 1, in the column called “Slow magnetic 1”. Figure 5 shows the bias estimation in *y* and *z*-directions. Observe that the abnormal behavior of the magnetic field affects the bias estimates of angular rate in all directions for QUKF and CF, which in turns does not happen with QRAUKF.

**Figure 6:**
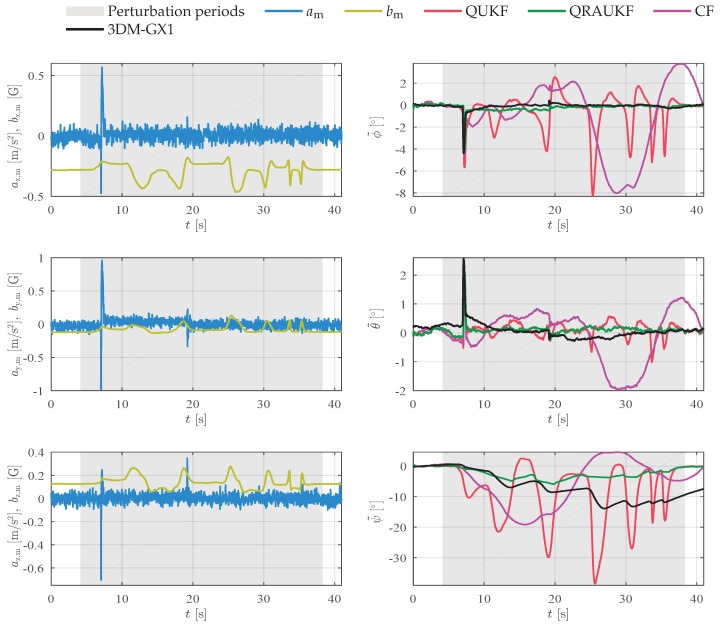
Results for slow magnetic disturbances experiment (third experiment), scenario (ii). In the left column, linear acceleration $a_{m}$ and magnetic field $b_{m}$ measurements, in the right column, the attitude error.

**Figure 7:**
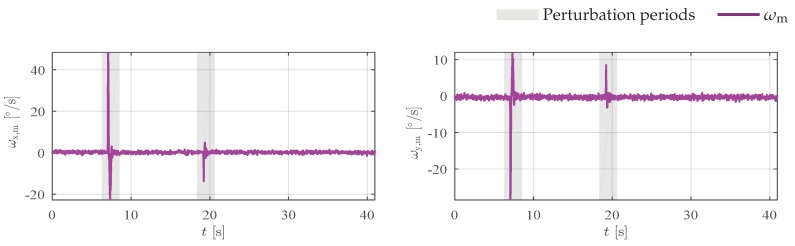
Angular rate $\omega_{y}$ and $\omega_{z}$, respectively, measured by the gyros for the third experiment, scenario (ii).

In a third experiment, the magnetic field was also artificially and slowly disturbed with magnetic material. Although similar to the previous experiment, in this one the magnetic field is continually disturbed during 30 s. Figure 6 shows the measurements and attitude estimation error for each axis. In addition to magnetic disturbance, during the experiment we accidentally touched the manipulator with the magnet and observed that the accelerometers and gyros also measure abrupt changes just before 10 s and 20 s. Figure 7 shows angular rate measurements for this experiment. All algorithms, except QUKF, are robust to outliers in acceleration and magnetic field measurements, however, as can be seen by the error results, all algorithms have large peak errors $\tilde{\phi }$ and $\tilde{\theta }$. These large errors are caused by the outliers in the angular rate measurements, that are not handled by the algorithms. In spite of these outliers, notice that QRAUKF shows the best result, as can be seen in Table 1, column “Slow magnetic 2”.

### 6.2. Linear Acceleration Disturbance

Roll ϕ and pitch θ angles are computed by the projection of the gravity vector, which is measured by the accelerometer. However, the accelerometer measures the linear body acceleration together with the gravity vector, which masks the gravity vector observation. Thus, the linear acceleration disturbs the observation of ϕ, θ, and consequently the heading angle ψ.

**Figure 8:**
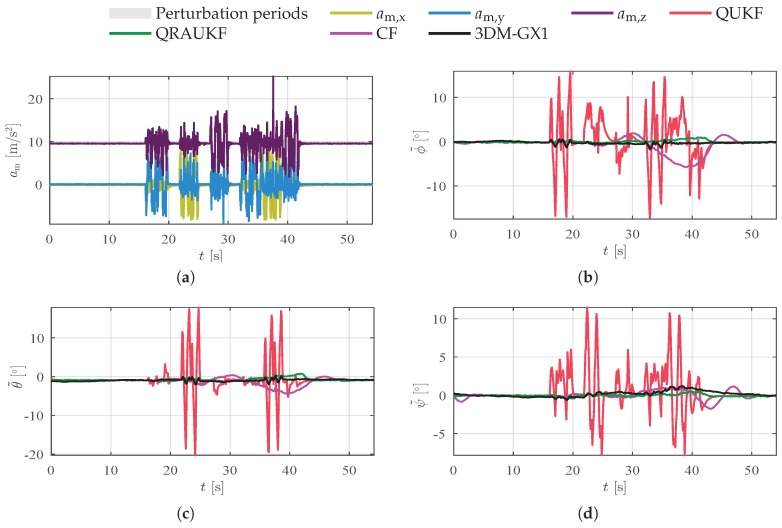
Results for linear acceleration disturbance experiment, scenario (iii). (**a**) shows the measured linear accelerations $a_{m}$; (**b**–**d**) show the estimation error for ϕ, θ and ψ angles, respectively.

To test the behavior of the algorithms against the perturbation of linear velocities, the manipulator executed independent translational movements in each axis. Figure 8a shows the accelerometer measurements. We observed that even when the movements are being executed separately in each axis, linear accelerations appear in all axes. This is probably due to a small angle in the link joining the IMU and the robot end effector. Figure 8b–d shows the attitude error. Table 2 shows the values of RMS for this and other experiments. Notice that the QUKF provides the worst results. In contrast, QRAUKF yields the best RMSE indexes and the smallest peak-error for heading angle. We also observe that after 30 s, the attitude error for QRAUKF grows due to the longer time exposed to linear acceleration perturbation. In fact, the QRAUKF estimates tend to converge to the value induced by the perturbed measurement after a period of time.

### 6.3. Rotations about the Origin

**Figure 9:**
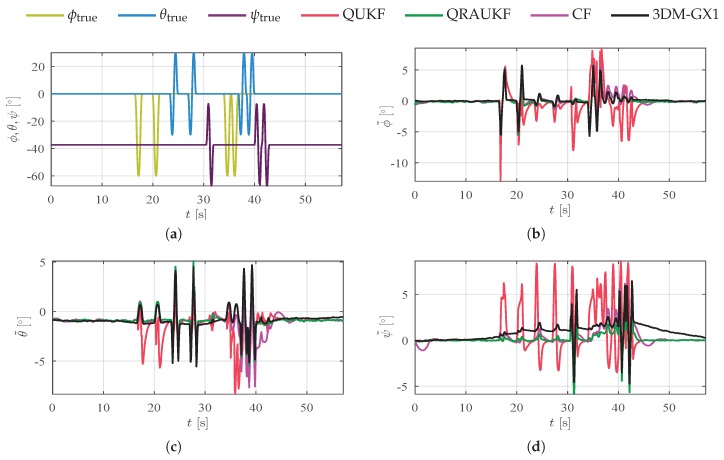
Results for individual axis rotation about the origin, scenario (iv). (**a**) shows actual orientation for individual axis movements; (**b**–**d**) show the estimation error for ϕ, θ and ψ angles, respectively.

**Figure 10:**
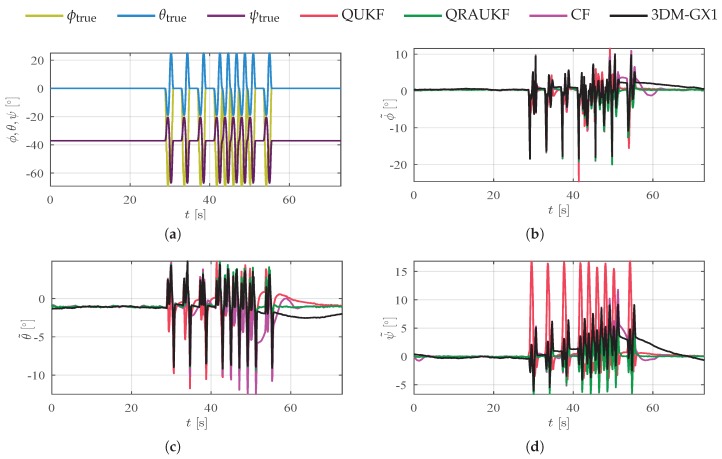
Results for simultaneous axes rotation about the origin, scenario (v). (**a**) shows actual orientation for simultaneous axes movements; (**b**–**d**) show the estimation error for ϕ, θ and ψ angles, respectively.

In our last two experiments, presented in Figure 9 and Figure 10, rotations about the origin in each axis separately and simultaneously were performed. In these cases, estimates are influenced by linear accelerations that appear due to a lever arm between the IMU and the robot end effector. Figure 9a and Figure 10a show the actual movement performed by the manipulator. Again, the proposed algorithm yields the best results, as shown by Table 2. Notice by Figure 9 that QRAUKF and 3DM-GX1 algorithm have similar errors, however, QRUKF converges faster to measurement after the perturbation finishes. The poor performance of KF and CF is due to bias estimates that are influenced by linear acceleration.

## 7. Conclusions

In this paper, a quaternion-based robust adaptive unscented Kalman filter for orientation estimation was presented. The algorithm ensures the unit norm of quaternion in all algorithm steps without forcing a normalization. The logarithmic map of unit quaternions is used to parametrize the error quaternion. This parameterization allows us to perform operations in Euclidean space and then use existing approaches to adapt the measurement covariance matrix and detect outliers. Due to the nonlinear nature of this transformation, unscented transform is used to compute the measured quaternion.

The proposed algorithm was compared to a nonadaptive version of UKF, a complementary filter, and commercial algorithm embedded in the IMU. Some experiments were performed to verify the performance of the algorithms in situations where distorted magnetic field and linear accelerations exist. The proposed algorithm shows the best RMSE results in all situations tested, and the smallest peak-error for linear acceleration disturbance. In addition, the proposed algorithm accurately estimates the gyros bias terms. Although in this paper we only show attitude estimation, the proposed methodology can be used along the standard UKF equations to estimate the full state vector of vehicles. The same ideas for disturbance rejection can also be extended for other vehicle states, once the sum and subtraction operations defined in Section 3.2 are the usual operations for Euclidean states.

## Figures and Tables

**Table 1:** Root Mean Square Error (RMSE) in degrees for disturbance scenarios (i) and (ii). The lowest RMSE results are highlighted in bold.

	Abrupt Magnetic			Slow Magnetic 1			Slow Magnetic 2		
**Algorithm**	$\tilde{\phi }$	$\tilde{\theta }$	$\tilde{\psi }$	$\tilde{\phi }$	$\tilde{\theta }$	$\tilde{\psi }$	$\tilde{\phi }$	$\tilde{\theta }$	$\tilde{\psi }$
*QRAUKF*	0.05	**0.04**	**0.07**	**0.07**	**0.09**	**1.84**	0.40	**0.19**	**3.11**
*QUKF*	0.05	0.05	0.20	0.98	0.51	13.0	1.66	0.27	12.52
*CF*	0.07	0.06	0.14	9.22	11.00	28.90	3.20	0.86	8.97
*3DM-GX1*	0.16	0.18	0.09	0.12	**0.09**	11.28	**0.35**	0.27	9.8

**Table 2:** Root Mean Square Error (RMSE) in degrees for disturbance scenarios (iii), (iv), and (v). The lowest RMSE results are highlighted in bold.

	Linear Acceleration			Individual Rotations			Simultaneous Rotations		
**Algorithm**	$\tilde{\phi }$	$\tilde{\theta }$	$\tilde{\psi }$	$\tilde{\phi }$	$\tilde{\theta }$	$\tilde{\psi }$	$\tilde{\phi }$	$\tilde{\theta }$	$\tilde{\psi }$
*QRAUKF*	**0.28**	**0.87**	**0.16**	**1.08**	**1.31**	**0.91**	**2.88**	**1.94**	**1.37**
*QUKF*	4.0	3.78	2.36	1.97	1.73	2.23	2.76	2.08	4.20
*CF*	1.87	1.60	0.53	1.17	1.61	1.17	2.97	2.54	2.03
*3DM-GX1*	0.37	0.99	0.39	**1.08**	1.34	1.37	**2.88**	2.17	2.06
